# Current status and innovative developments of CAR-T-cell therapy for the treatment of breast cancer

**DOI:** 10.1186/s12935-024-03615-8

**Published:** 2025-01-04

**Authors:** Hany E. Marei, Khaled Bedair, Anwarul Hasan, Layla Al-Mansoori, Sara Caratelli, Giuseppe Sconocchia, Alice Gaiba, Carlo Cenciarelli

**Affiliations:** 1 Department of Cytology and Histology, Faculty of Veterinary Medicine, Mansoura University, Mansoura, 35116 Egypt; 2 Department of Social Sciences, College of Arts and Sciences, Qatar University, P.O. Box 2713, Doha, Qatar; 3 Department of Mechanical and Industrial Engineering, College of Engineering, Qatar University, Doha, Qatar; 4 Biomedical Research Center, Qatar University, P.O. Box 2713, Doha, Qatar; 5 Institute of Translational Pharmacology-CNR, Rome, Italy

**Keywords:** CAR T cells, Breast cancer, CAR signaling, T cell persistence, T cell exhaustion, In vivo studies

## Abstract

Breast cancer will overtake all other cancers in terms of diagnoses in 2024. Breast cancer counts highest among women in terms of cancer incidence and death rates. Innovative treatment approaches are desperately needed because treatment resistance brought on by current clinical drugs impedes therapeutic efficacy. The T cell-based immunotherapy known as chimeric antigen receptor (CAR) T cell treatment, which uses the patient’s immune cells to fight cancer, has demonstrated remarkable efficacy in treating hematologic malignancies; nevertheless, the treatment effects in solid tumors, like breast cancer, have not lived up to expectations. We discuss in detail the role of tumor-associated antigens in breast cancer, current clinical trials, barriers to the intended therapeutic effects of CAR-T cell therapy, and potential ways to increase treatment efficacy. Finally, our review aims to stimulate readers’ curiosity by summarizing the most recent advancements in CAR-T cell therapy for breast cancer.

## Introduction

Breast cancer, the most common type of cancer in women, is characterized by the progressive dysplasia of malignant cells in the ductal or lobular region of the breast [1]. Despite continuous advancements in therapy, there are still insufficient options for breast cancer treatment [2]. Among these is triple-negative breast cancer (TNBC), the most aggressive type with the worst prognosis, making up 15–25% of all cases of the disease [3, 4]. Human epidermal growth factor receptor 2 (HER-2), progesterone receptor (PR), estrogen receptor (ER), and Ki-67 expression have made it possible to categorize breast cancers into four groups: TNBC (HER2-/ER-/PR-), Luminal A (HER2-/ER+/PR +, low proliferation), and Luminal B (HER2-/ER+/PR+, high proliferation). Treatments for HER2-positive breast cancer include chemotherapy, endocrine therapy, tyrosine kinase receptor inhibitors (TKIs) like lapatinib, and anti-HER-2 therapy like trastuzumab [5, 6]. Unfortunately, because specific targets are absent, endocrine treatment and HER-2 targeted therapy have a limited response in TNBC. Chemotherapy (anthracycline + taxane) is the main treatment for TNBC; however, long-term recurrence and treatment resistance still need to be addressed [7]. Notably, TNBC frequently reacts favorably to chemotherapy; however, relapses are more common in the first three years after diagnosis [4, 8], indicating an unfulfilled need for innovative, effective treatments.

**Fig. 1:**
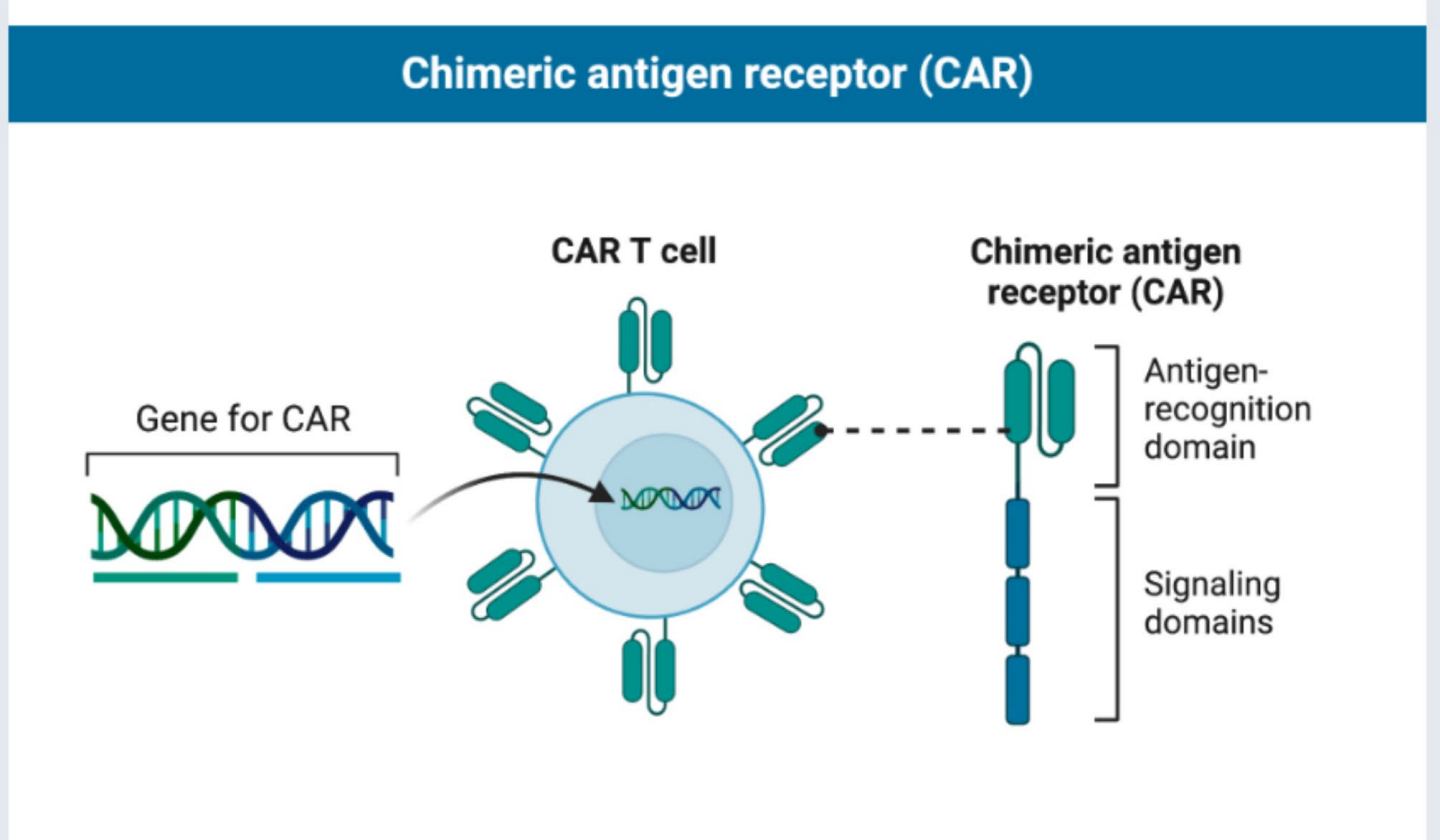
Chimeric Antigen Receptor (CAR) T cell Therapy. The extracellular domain of CARs uses the scFv, which is derived from the variable region of antibodies, to identify tumor antigens on the surface of tumor cells. A transmembrane (TM) domain connects the extracellular and intracellular regions of CARs, as well as one or more costimulatory domains that cause a longer T-cell activation and cytokine-mediated killing of tumor cells

Chimeric antigen receptor (CAR) T cells, or genetically modified T cells, have emerged as a promising therapeutic option for several cancers. CAR T cells are designed to specifically target antigens to cancer [9, 10]. CAR T-cell functionality is influenced by various domains present in CARs, including a transmembrane (TM) domain that links the extracellular and intracellular portions, and one or more costimulatory domains to induce a prolonged T-cell activation [11] (Fig. 1). The CD3ζ stimulatory domain present in the majority of CAR architectures currently phosphorylates its three immunoreceptor tyrosine-based activation motifs (ITAM) to activate T cells [12](16). Nevertheless, current studies indicate that the three ITAM high degree of phosphorylation may be redundant, reducing their in vivo durability [13, 14]. Furthermore, the safety of therapy has been impacted by cytokine release syndrome (CRS), which is caused by excessive cytokine secretion, and serious negative consequences due to insufficient target antigens. Furthermore, has been shown in numerous studies that lack of sustained in vivo persistence was detrimental to the treatment of solid tumors (21) and, may hinder the achievement of permanent remissions in hematological malignancies [15, 16].

Recent research has demonstrated that CAR-containing exosomes express cytotoxic molecules at high levels and inhibit the growth of tumors, indicating that these exosomes are a relatively safe alternative to CAR-T therapy without the acute toxicities associated with lung cancer [17, 18]. Endogenous signaling molecule activating (ESMA) CAR is an alternative architecture that Ebbinghaus et al. developed to increase CAR T cell persistence for TNBC treatment. It is made up of TM domains generated from CD335, CD336, or CD64, which interact with DAP12, the FcRγ-chain, and endogenous signaling molecules such as DAP12 and CD3ζ. Compared to second-generation CAR, EGFR-directed ESMA CAR T cells showed a persistent death of TNBC cell line MDA-MB-231 at a slower velocity. Moreover, there was a decrease in the expression of the exhaustion marker and cytokine secretion, a strong tumor infiltration, and improved memory-like behavior. An in vivo xenograft mouse model demonstrated significant anti-tumor activity for the main candidate CD335 ESMA CAR [19].

This review focuses on the use of CAR-based immunotherapy in the management of BC, building on the success of CAR therapy in the treatment of tumors. The purpose of this work is to offer new viewpoints and recommendations for further research on BC care. The development of antigen-specific CAR-T cells and their uses in fundamental research and clinical trials are examined after a description of tumor antigens in breast cancer as well as their therapeutic relevance. We hope that this review will provide a useful update on the application of CAR-T cells in the treatment of breast cancer, along with recommendations for enhancing therapeutic efficacy [20].

## Development and mechanism of action of CAR-T cells

**Fig. 2:**
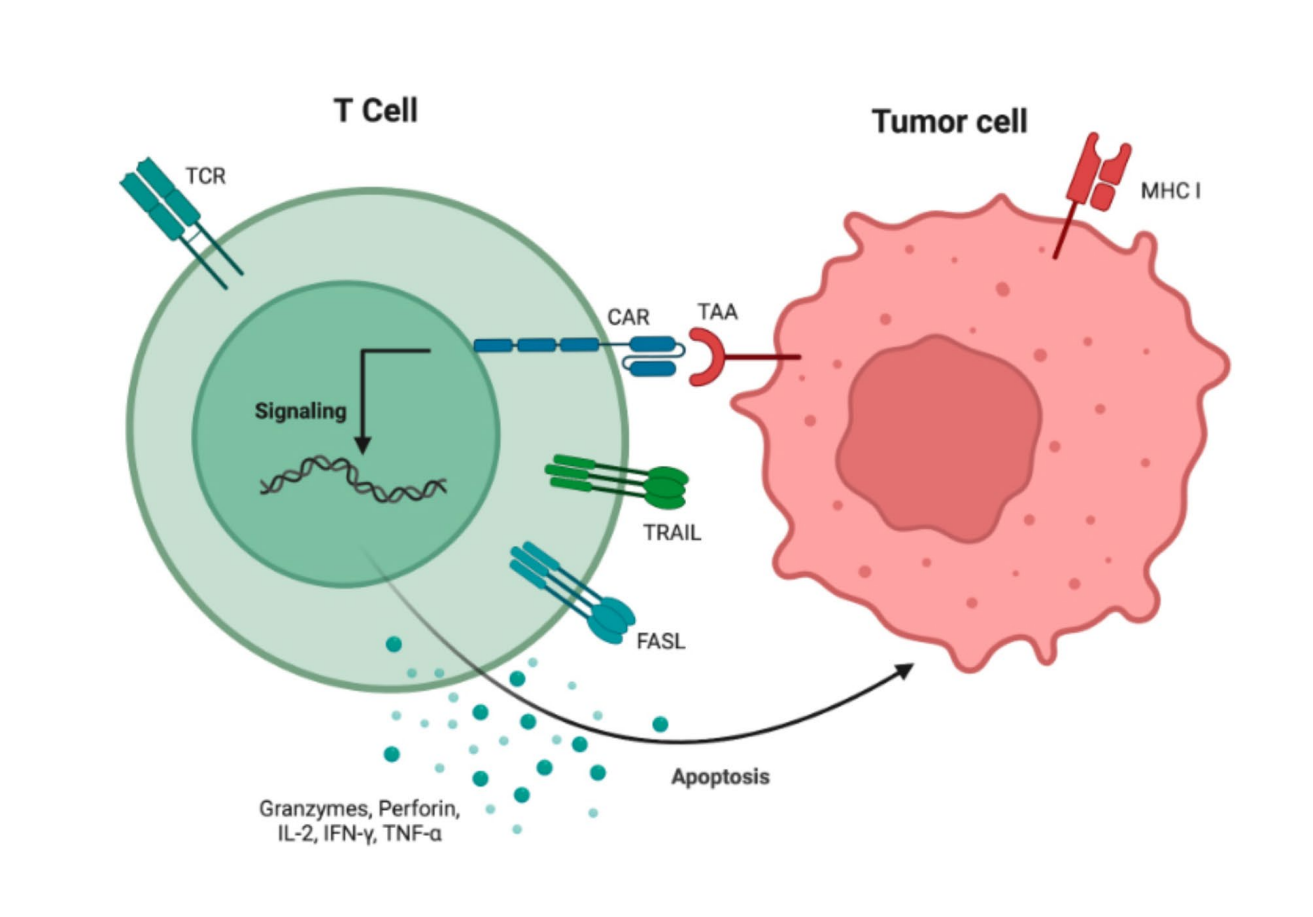
CAR T cell causing cancer cell death. The interaction of TAA with the CAR results in the production of granzymes, perforin, IL-2, INF-α, and TNF-α, which cause tumor cells to undergo apoptosis. This interaction occurs independently of the MHC I antigen

Chimeric antigen receptor (CAR) T cell therapy was developed using autologous peripheral blood to separate patients’ T cells, which are then engineered ex vivo to express synthetic receptors that can recognize TAAs. After the cells are expanded outside the body, patients receive reinfused CAR-T cells as a cancer treatment [21, 22]. The scFv derived from the variable region of antibodies, is used by the extracellular domain of CARs to recognize tumor antigens. When CAR-T cells come into contact with tumor cells, they can recognize antigens on their surface (Fig. 2). The first generation of CAR-T cell treatment produced unsatisfactory clinical results because the CAR-T cells failed to expand and exhibit low persistence [23, 24]. In further engineering, costimulatory signaling domains were added to CARs to solve these issues. In contrast to their previous generations, CARs of the second generation incorporate an additional costimulatory domain (such as CD28, 41BB, or ICOS) [25]. To increase T cell survival and cytotoxic potential, two additional costimulatory domains (such as CD27, CD28, 41BB, ICOS, and OX-40) were added to the third generation of CARs [26, 27]. The addition of a nuclear factor of activated T cells (NFAT) domain carrying an inducible IL-12 cassette initiated the fourth generation of CARs, also referred to as T cells redirected for universal cytokine-mediated killing (TRUCK) [28]. IL-12 is released and accumulates in the targeted region upon recognition of tumor antigens by CAR-T cells and consequently, NK cells and macrophages are attracted to the tumors to eradicate the cancer cells [29, 30]. The fifth generation of CARs is produced by combining IL-2 receptor β-chain fragment (IL-2Rβ) with the second generation of CARs. The IL-2Rβ fragment’s binding site can initiate the JAK-STAT signaling pathway, resulting in full T-cell activation and enhanced persistence [31, 32].

### Targets antigens investigated for the CAR-T therapy of TNBC

Since many tumor-associated antigens (TAAs) are also expressed on healthy tissues, increasing the possibility of off-target effects and serious toxicity, antigen selection is a crucial challenge in the development of CAR-T-cell treatments for breast cancer. To prevent harming important organs, the ideal target antigens would be substantially expressed on malignant cells and nonexistent or barely present on healthy tissues. Low amounts of common antigens investigated for breast cancer, including HER2, MUC1, and EGFR, are also present in normal tissues, raising the possibility of unintentional CAR-T-cell activation and toxicities such “on-target, off-tumor” consequences. Furthermore, the heterogeneous tumor microenvironment’s dynamic antigen expression introduces an additional layer of complication since CAR-T cells may come across variants of antigen deletion or variable antigen levels, which would decrease the effectiveness of treatment. Researchers are investigating techniques including dual-target CARs, tumor-restricted promoters, and enhanced safety switches to increase safety and specificity. These approaches may provide more accurate targeting capabilities and reduce off-target dangers [33].

**Fig. 3:**
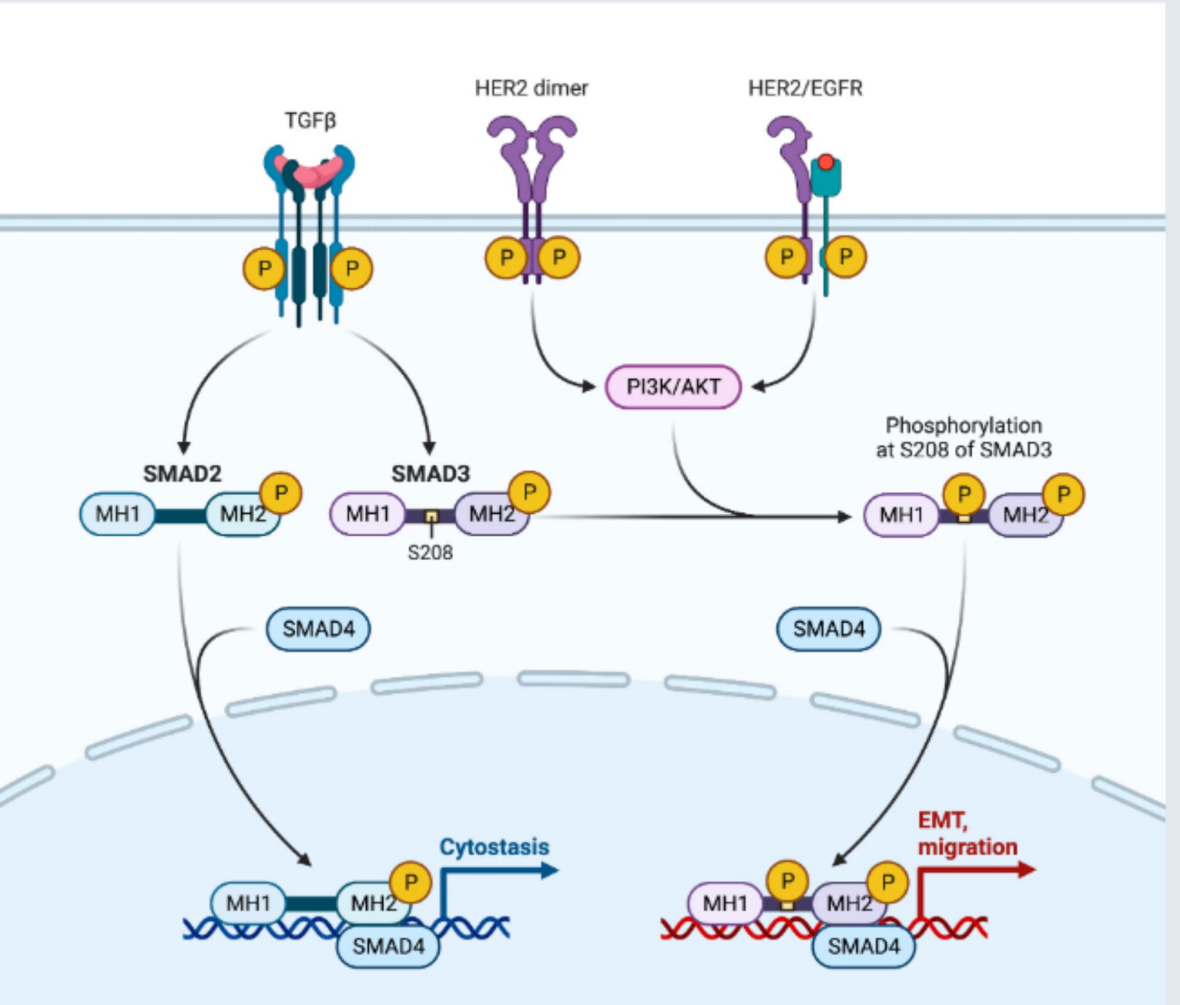
Downstream pathways activation of TNBC tumor associated with TGFβ, and HER2/EGFR. The stimulation of downstream signaling pathways, including PI3K/AKT, Ras/MEK/ERK, PLCγ/PKC, and JAK/STAT, is a crucial factor in the development of tumors [42]. Cell migration, survival, proliferation, and death are all regulated by the PI3K/AKT signaling system. Cell survival, migration, and proliferation are governed by the pathways of Ras/MEK/ERK and PLCγ/PKC, whereas the JAK/STAT system governs angiogenesis and metastasis. HER2 and EGFR are two RTK that have been linked to abnormal expression or hyperactivation in breast cancer [42]. Because HER2 signaling is triggered by somatic mutations in the HER2 gene, these mutations also aid in the development of breast cancer

After being triggered by growth factors or hormones, receptor tyrosine kinases (RTK) regulate essential cellular processes such as proliferation, differentiation, metabolism, and survival [34]. A key component in the formation of tumors is the activation of downstream signaling pathways, such as PI3K/AKT, Ras/MEK/ERK, PLCγ/PKC, and JAK/STAT [35]. The signaling pathway PI3K/AKT controls apoptosis, proliferation, survival, and migration of cells. The JAK/STAT system controls angiogenesis and metastasis, whereas the pathways of Ras/MEK/ERK and PLCγ/PKC are responsible for cell survival, migration, and proliferation. Two RTK that have been connected to aberrant expression or hyperactivation in breast cancer are HER2 and EGFR [35] (Fig. 3). In this section, the primary targets of CAR-T cell therapy are five RTK. The receptor tyrosine-protein kinase (RTK) family’s HER/ERBB family includes human epidermal growth factor receptor 2 (HER2), also known as ERBB2 [36]. Tumor metastasis initiates with the activation of several downstream signaling pathways that promote the expression of genes encoding the epithelial-mesenchymal transition (EMT) [37–39]. HER2 gene amplification or overexpression, which affects 20–30% of patients with breast cancer, is associated with a poor prognosis, worse clinical outcomes, and the advancement of the disease [40, 41]. Because HER2 signaling is triggered by somatic mutations in the HER2 gene, these mutations also aid in the development of breast cancer (Fig. 3) [42]. Therefore, therapies for breast cancer may target HER2. The FDA approved trastuzumab, the first targeted treatment for breast cancer. Additionally, clinical outcomes have improved with additional monoclonal antibodies that target HER2 [43]. Targeting HER2 + malignancies, HER2-CAR-T cells revealed a substantial reduction in tumor growth [44] and a regression in brain tumor metastasis [45] in preclinical investigations. Furthermore, long-term survival was increased in xenograft mouse models created from the trastuzumab-resistant JiMT-1 cell line when HER2-CAR-T cells invaded the tumor matrix and removed the solid tumor [46]. Furthermore, HER2-targeted CAR-T cells led to tumor remission even at lower dosages and generated a strong immune response. These findings imply that CAR-T cell therapy for breast cancer may target HER2.

EGFR, or the epidermal growth factor receptor, is also HER1, sometimes referred to as ERBB1, and is a member of the ERBB family. After ligand binding activates and causes the same signaling pathways downstream of HER2 [42]. EGFR overexpression has been linked to larger tumor sizes and worse clinical outcomes at diagnosis in 15–30% of cases of breast cancer patients [47, 48]. Notably, TNBC, an estrogen-positive subtype of the disease that is PR-negative, and HER2-negative, accounts for approximately 45–70% of all cases of TNBC [49]. TNBC is also known to overexpress EGFR. Consequently, several EGFR-targeted therapies have been investigated for the treatment of TNBC, including CAR-T therapy showing anticancer and cytotoxic effects in vitro and in vivo [49–51].

CAR T cell-based therapy for breast cancer also targets RTK, including AXL, hepatocyte growth factor receptor (HGFR), and receptor tyrosine kinase-like orphan receptor 1 (ROR1). These RTK play similar functions in the pathophysiology of BC.

The RTK family also includes receptor tyrosine kinase-like orphan receptor 1 (ROR1). ROR1 expression is highest during embryogenesis, decreases during fetal development, and finally vanishes in tissues that have undergone terminal differentiation [52]. Notably, a few malignant tumors, such as breast cancer, have high expression levels of ROR1 as well [53, 54]. Increased expression of ROR1 in breast cancer leads to the development of ABCB1, an ATP-dependent drug efflux pump that increases tumor recurrence and resistance to chemotherapy [55]. Remarkably, chemoresistance could be reversed with efflux pump inhibitors or antibodies specific to ROR1 [56]. ROR1-CAR-T cell application has proven the release of cytokines and cytolytic activities that aid in tumor destruction [57].

AXL belongs to the RTK family, specifically TAM [58]. AXL primarily transmits signals for metastasis, increases chemo-resistance, and promotes survival rather than acting as a catalyst to initiate malignant transformation [59]. After activation, downstream signaling pathways like PI3K/AKT, MAPK, and JAK/STAT are stimulated by AXL autophosphorylation, which in turn regulates the functions of cancer cells [60]. AXL is thought to be a marker of TNBC since it is significantly expressed in TNBCs in comparison to all other breast cancers [61]. AXL overexpression is a reliable indicator of poor clinical and survival outcomes [62]. It has been demonstrated that the ATP-competitive inhibitors of AXL inhibit tumor growth in animal models by causing BC cells to undergo apoptosis [63]. In vitro, third-generation AXL-CAR-T cells exposed to AXL-positive cancer cells showed anti-tumor effects through the induction of cytokine production and response to cell death [64, 65]. Furthermore, large cytotoxic effects were observed in vitro and decreased tumor size in MDA-MB-231-derived xenograft mouse models using a novel combination strategy that combined constitutive active IL-7 receptor inhibition with AXL-CAR-T [66].

Cell surface proteins on tumor cells act as tumor antigens to identify CAR-T cells and reinforce the anticancer properties of T cells. Eleven surface proteins whose expression is abnormally elevated in breast cancer may benefit from CAR-T cell therapy. These include mucin 1 (MUC1), mesothelin (MSLN), CD70, CD133, CD44 containing variant exon v6 (CD44v6), intercellular adhesion molecule-1 (ICAM1), trophoblast cell surface protein 2 (TROP2), tumor endothelial marker 8 (TEM8), epithelial cell adhesion molecule (EpCAM), chondroitin sulfate proteoglycan 4 (CSPG4), tumor endothelial marker 8 (TEM8), and folate receptor alpha (FRα). It has been reported that MUC1 overexpression occurs in nearly 90% of breast tumors [67].

Overexpression of MUC1 inhibits adhesion [68] while activating downstream signaling pathways like ERK1/2 and NFκB [69] to control tumor migration and development. Clinically, MUC1 overexpression in breast cancer patients is associated with advanced tumor stages and a poor prognosis [70]. Mesothelin (MSLN) [71] overexpression has been shown to occur in a number of solid tumors, including breast cancer, particularly in more aggressive and advanced subtypes of the disease. Patients with breast cancer who overexpress MSLN are more likely to experience poorer clinical outcomes and develop chemotherapy resistance. The constitutive activation of intracellular signaling pathways (NF-kB, PI3K, and MAPK) by MSLN overexpression facilitates the formation and progression of tumors [72]. Thus, targeting MSLN could be one strategy for cancer immunotherapies.

Even though multiple solid tumors have been reported to overexpress CD70, lymphoid tissues are the only tissues that express CD70 [73, 74]. CD70 regulates lymphocyte differentiation, cell survival, and growth after binding CD27 [75]. There is debate over CD70’s involvement in breast cancer (85). Preclinical and clinical research on a variety of immune treatment approaches, including monoclonal antibodies and CAR-T cells that target aberrantly CD70 have yielded positive results [75].

One biomarker seen on the surface of CSCs is called CD133, which is thought to be the most accurate indicator of malignant precursors in a variety of solid tumors, including breast cancer [76]. Additionally, in patients with breast cancer, there is an enhanced expression of CD133, which is connected with a poor prognosis and cancer progression [77]. These characteristics point to CD133 as a possible immunotherapy target [78]. Indeed, CD133-targeted treatments demonstrated remarkable tumor suppression potential in several solid tumors. By inhibiting the growth and recurrence of the tumor in MDA-MB-231 xenograft models, the combination of paclitaxel and anti-CD133 antibodies greatly enhanced the therapeutic effects [79]. This was in contrast to the group that received paclitaxel alone.

CD44v6 activates PI3K/Akt and MAPK signaling pathways that regulate cell invasion, apoptosis, and metastasis [80]. BC have been demonstrated upregulation of CD44v6, particularly in invasive breast cancer cell lines [81]. Tumor cell invasion and migration were significantly suppressed by microRNA-mediated downregulation of CD44v6 (90). Additionally, a meta-analysis showed a relationship between advanced histological stages, lymph node metastases, and a poor overall survival rate in cases of breast cancer associated with the overexpression of CD44v6 [82].

Several trials have demonstrated the potential effectiveness of targeting Epithelial Cell Adhesion Molecule (EpCAM) in the treatment of solid tumors. Antibodies that targeted EpCAM exhibited anticancer properties. Adecatumumab, for instance, inhibited BC metastasis in a dose- and target-dependent way. As consequence, catumaxomab, which targets EpCAM, is already licensed for the treatment of cancer. In a mouse model using transplanted TNBC cells, a cytolytic fusion protein that targets EpCAM proved remarkable tumor-inhibiting activity [83].

A significant impact on chemoresistance and cancer progression is caused by overexpression of CSGP4, which shortens the time to recurrence (TTR) and lowers overall survival (OS) [84]. A meaningful target for cancer immunotherapy is CSGP4. TNBC cells co-cultured with anti-CSGP4 monoclonal antibody, decreased tumor growth and metastasis and reduced cell migration and expansion of TNBC cell-derived transplants in immunodeficient mice [85].

Numerous cancers, particularly breast cancer, have been linked to ICAM1 overexpression [86]. There was evidence that TNBCs had higher levels of ICAM1 mRNA and proteins when compared to normal breast tissues and other subtypes of breast cancer. Cell invasion and migration in highly metastatic MDA-MB-435 cells were remarkably suppressed when ICAM1 was blocked by antibodies [87]. ICAM1 appears to be a potential therapeutic target as a result.

Its overexpression on the tumor vasculature’s epithelial cells and its involvement in tumor angiogenesis led to its identification [88](97). Elevated TEM8 expression in breast cancer has been linked to an increased risk of tumor relapse (98). Tumor growth and metastasis were inhibited by antibodies that blocked TEM8/ANTXR1 or TEM8/ANTRX1 knockdown genetically [88].

Tumor-associated calcium signal transducer 2 (TROP2) overexpression is related with poor clinical outcomes, including disease progression and a short life expectancy time. Targeting TROP2 may therefore be beneficial when treating tumors that are TROP2-positive. Sacituzumab govitecan targeting TROP2, an antibody-drug conjugated, was recently approved by the FDA to treat metastatic TNBC that has relapsed or is refractory [89]. Human antibodies that target TROP2 have anticancer properties both in vivo and in vitro by blocking signaling molecules that are essential for cell survival [90].

Overexpression of Folate Receptor alpha (FRα) in BC is associated with unfavorable clinical outcomes including a shorter TTR and OS [91]. When FRα is overexpressed on tumor cells, it becomes a desirable target for treatment.

A very small fraction of GD2-overexpressing cancer cells were able to grow into mammospheres and cause tumors in mice. In vivo tumor spread and tumor growth were completely reversed by inhibiting GD3S, a crucial enzyme involved in the manufacture of GD2 [92]. Additionally, the proto-oncogene cMET was constitutively activated by the overexpression of GD2, which led to increased tumor development, metastasis, and proliferation [93]. All of these facts point to GD2 as a potential anticancer target. Both in vivo and in vitro investigations demonstrated the powerful inhibition of breast cancer that dinutuximab targeting GD2 might provide [94]. By obstructing the mTOR pathway, it prevented breast cancer cells from adhering to one another, migrating, and forming mammospheres [95].

NKG2D controls survival, cytotoxicity, and cytokine production [96]. In the tumor microenvironment, NKG2D ligands are significantly expressed by immune system cells, cancer cells, and infected cells [97]. NKG2D ligand expression in breast cancer patient samples demonstrated that it was only expressed in cancer cells [98]. Undoubtedly, cancer immunotherapy may target NKG2D ligand. A study that used miRNA to silence NKG2D ligand in vitro revealed that a decrease in NKG2D ligand increased NK cell-mediated cytotoxicity.

One of the most widely utilized serum tumor indicators for metastatic breast cancer is carcino-embryonic antigen (CEA). It usually indicates poor OS, disease-free survival (DFS), metastasis of lymph nodes, larger tumor size, and advanced TNM stage [99]. A higher degree of CEA expression suggests a greater chance of antitumor effects from the CEA-targeting strategy.

An in silico analysis was performed to compare patterns of gene expression to find possible targets for immunotherapy against breast cancer. This led to the discovery of 36 putative tumor-surface antigens, such as integrin beta-6 (ITGB6), fibroblast growth factor receptor-4 (FGFR4), and ectonucleotide pyrophosphatase/phosphodiesterase 1 (ENPP1) [100]. Further research is required to determine the potential of those potential targets as not much has been done to elucidate their therapeutic benefits.

## Application of CAR T cell for BC immunotherapy

While Luminal A, Luminal B, and HER-2-positive BC can all be effectively treated, Current research has reported positive CAR-T therapy outcomes in Luminal A type. The treatment of HER-2-positive BC with CAR-T therapy has shown outstanding results [101]. There is, however, little data supporting CAR-T treatment for Luminal B type. A breakthrough has been made in the treatment of TNBC using CAR-T therapy [102]. The applicability of CAR-T treatment in various breast cancer subtypes is summarized in the sections that follow, with an emphasis on TNBC’s pertinent targets.

### Luminal A and luminal B

Luminal A (HER2-/ER+/PR+, low proliferation) and luminal B (HER2-/ER+/PR+, high proliferation) are two subtypes of breast cancer with a better prognosis when compared to other forms [103]. Despite the encouraging clinical outcomes of endocrine therapy and chemotherapy, CAR-T therapy is still being studied as a possible treatment for Luminal A. A considerable percentage of patients with the luminal A subtype, for instance, express the tumor-associated antigen ganglioside GD2 [104]. According to Seitz et al., GD2-targeted CAR-T cells exhibited significant cytolytic activity against the GD2-positive Luminal A cell line MCF 7, but low tumor activity against the GD2-positive Luminal A cell line T-47D [105]. However, Zhang et al. found that MSLN-specific CAR-T cells were able to kill MSLN-positive MCF 7 breast cancer cells and release cytokines [106]. According to Bajor et al., MCF-7 cells with low PD-L1 were killed by PD-L1-CAR-T cells; however, PD-L1-CAR-T cells plus HER-2-CAR-T cells boosted PD-L1 expression in MCF-7 cells, accelerating the killing process [107]. AXL, B7-H4, EGFR, FcγRI (CD64), HER2, and MCF 7 and SK-BR-3 cell lines are among the targets of CAR-T cells; they also exhibit antitumor effects on these lines [108]. However, fewer studies have been done on CAR-T treatment for Luminal B tumors. Consequently, more study on CAR-T cells is needed to treat Luminal A and Luminal B subtypes.

The tumor microenvironment (TME) of Luminal A and Luminal B breast cancers is more immunosuppressive than that of other subtypes, such as triple-negative breast cancer (TNBC). This can make immunotherapies, such as checkpoint inhibitor and CAR-T-cell therapies, less effective. In contrast to TNBC, which is recognized for having a higher mutational burden and a greater presence of immune cells, Luminal A and Luminal B tumors generally express hormone receptors (estrogen and/or progesterone), but they also exhibit lower levels of immune cell infiltration. Tumor-associated macrophages (TAMs), myeloid-derived suppressor cells (MDSCs), and regulatory T cells (Tregs) frequently dominate the immunosuppressive TME in luminal tumors. These cells release cytokines and other substances that prevent immune activation and encourage tumor growth. The efficacy of checkpoint inhibitors is also diminished in these malignancies due to decreased expression of immune-activating factors such PD-L1. On the other hand, checkpoint blockage and CAR-T cells thrive in the more inflammatory TME of TNBC. Combination therapy, including adding immune modulators or focusing on particular immunosuppressive pathways, may improve CAR-T-cell efficacy and encourage a more positive immune response in order to get beyond these obstacles in Luminal subtypes [109].

The efficiency of immunotherapeutic approaches, such as checkpoint inhibitors and CAR-T-cell therapy, may be limited by the generally lower levels of immune cell infiltration, especially T cells, seen in luminal A breast cancers. Compared to more inflammatory subtypes like triple-negative breast cancer (TNBC), these cancers are less susceptible to immune-based therapies because they exhibit relatively modest immune activation despite frequently having high expression of estrogen and progesterone receptors. The tumor microenvironment is less active and does not successfully support anti-tumor immunity as a result of the limited infiltration of immune cells, particularly cytotoxic T cells. However, Luminal B tumors may have a more active immunological milieu and a moderate immune cell infiltration, including larger amounts of CD8 + T cells, while being hormone receptor-positive as well. Their unique biological traits, such as higher proliferative indices and more tumor heterogeneity, which can aid immune escape mechanisms, nonetheless present difficulties for them. Notwithstanding these obstacles, T-cell infiltration-boosting techniques such immune checkpoint blockade or combination therapy may enhance therapeutic outcomes in both Luminal A and Luminal B subtypes [110].

Finding appropriate targets for CAR-T-cell therapy and other immune-based treatments is more difficult in Luminal A and Luminal B breast cancer subtypes because the expression of particular tumor-associated antigens (TAAs) is frequently less prominent in these subtypes than in more aggressive subtypes like triple-negative breast cancer (TNBC). Although the overexpression of hormone receptors (progesterone and estrogen) is a common characteristic of these subtypes, they frequently show lower levels of tumor-associated antigens, such HER2, which are more frequently targeted in treatments for other subtypes of breast cancer. The effectiveness of CAR-T-cell therapies, which depend on identifying and focusing on certain molecules on the surface of tumor cells, is restricted by the decreased expression of these antigens. Furthermore, it is challenging to create efficient CAR-T cells that can specifically target and destroy tumor cells without producing off-target effects due to the lack or poor expression of these antigens. Strategies including the discovery of new tumor-specific antigens, the application of bispecific T-cell engagers, or combination treatments incorporating immune modulators may be required to overcome this obstacle and improve the efficacy of immunotherapies in Luminal A and Luminal B malignancies [111].

The elevation of immune checkpoint molecules like PD-L1 is one of the most notable resistance mechanisms that both Luminal A and B breast cancer subtypes may develop, which drastically lowers the efficacy of immunotherapy. These mechanisms are frequently brought on by the tumor’s capacity to adapt and elude immune surveillance, which reduces the response to immune-based therapies like as checkpoint inhibitors and CAR-T-cell therapy. A lower initial immunological response may result from the low immune cell infiltration commonly observed in Luminal A tumors; nevertheless, immune evasion may be exacerbated by the overexpression of checkpoint molecules, such as PD-L1. Similarly, Luminal B cancers may express higher amounts of PD-L1 in response to immunological pressure, even if they show more immune infiltration. This can restrict the therapeutic efficacy of immune checkpoint blockage and impede the activity of effector T cells. One of the main obstacles to treating these subtypes is the development of resistance through immune checkpoints, which calls for methods to block these pathways. For example, combining immune checkpoint inhibitors with CAR-T-cell therapy or other immunomodulatory drugs to improve tumor clearance [112].

Because of the inherent heterogeneity within Luminal A or B breast cancer subtypes, it is difficult to find trustworthy biomarkers to predict which individuals would respond well to immunotherapy. Although they are typically thought of as hormone receptor-positive, luminal A and B cancers have unique genetic and immunological traits that can differ greatly from patient to patient. Because variables such tumor infiltrating lymphocytes (TILs), immune checkpoint molecule expression (e.g., PD-L1), and mutation burden can vary within the same subtype, this heterogeneity makes it more difficult to define consistent criteria for patient selection. Certain Luminal B cancers, for instance, might be more sensitive to immunotherapies due to increased immune infiltration or higher PD-L1 expression, whilst other tumors might have less immunological activation and be less likely to respond favorably to such therapies. Although their usefulness in Luminal A and B malignancies is still being studied, the use of biomarkers such tumor mutational burden (TMB), TILs, or gene expression profiles may aid in response prediction. As the field develops, creating reliable, repeatable biomarkers that can precisely classify patients according to their propensity to benefit from immunotherapy will be crucial for enhancing treatment results and customizing therapy [113].

### HER2-positive breast cancer

The use of CAR-T cells that specifically target HER2 to eradicate HER2-positive breast tumors has advanced significantly. HER2-targeted CAR-T cells block tumor growth in vivo and in vitro in HER2-positive tumor cells [114]. Comparably, HER2-positive cancer cells like SK-BR-3 and MCF-7 cells are effectively lysed by CAR-T cells transduced with trastuzumab scFv [17]. HER2-targeted CAR-T cells had a potent anti-tumor effect on HER2-positive breast cancer when coupled with anti-PDL1 treatment [115]. However, normal lung cells that express HER2, HER2 targeted CAR-T cells induce in multi-organ failure [116]. HER2-targeted CART cells have trouble being effective in the therapy of breast cancer due to their off-target toxicity. Consequently, increasing the therapeutic efficiency of HER2-targeted CAR-T cells is the main goal of research for the treatment of HER2-positive breast cancer. Bispecific CAR-T cell therapy for HER2-positive breast cancer has made significant progress. Bispecific CAR-T cells which recognize both HER2 and melanocytic protein (gp100), for instance, may remove orthotopic mammary tumors that express HER2 in the brain and breast of immunocompetent animals [117].

Bispecific CAR-T cells demonstrated cytotoxic efficacy against MUC 1 and HER2 in breast cancer [118]. A cutting-edge therapeutic option is provided by CAR-T cells with multitargeting capabilities, which carry several distinct CARs to improve tumor cell targeting [119].

TanCAR-T cells are a type of unique bispecific CAR that can recognize several tumor antigens with a single CAR-T cell. They are composed of two scFV domain junctions. When recognized concurrently, the two distinct CARs of TanCAR-T cells can boost T cell activation synergistically. For instance, the anticancer impact on glioblastoma is enhanced by tandem CAR-T targeting HER2 and IL13Ra2 [120]. Targeting both CD19 and HER2, tanCAR cells lyse target cells that are either CD19- or HER2-positive while also secreting IL-2 and IFN-γ at the same time. TanCAR subunits can move almost freely on tandem CAR-T cells thanks to their two unique CARs, which also improve tandem recognition [121](126). TanCAR cells have so demonstrated a significant deal of potential for the treatment of HER2-positive breast cancer.

### TNBC

30% of mortality from breast cancer is attributed to TNBC [47]. Compared to other subtypes of breast cancer, TNBC has a higher histological grade, is highly invasive, and has distant metastases [122]. Following curative surgical resection, postoperative adjuvant chemotherapy is the primary treatment for TNBC [123]. Nonetheless, TNBC’s poor prognosis and high recurrence rate continue to be difficult to manage. Targeted therapy is insensitive while treating TNBC because there aren’t any obvious targets. For TNBC, chemotherapy regimens that include paclitaxel and anthracyclines are utilized. On the other hand, severe side effects and toxicity do not increase patient survival [124]. The FDA’s recent approval of PD-1 inhibitors, PARP inhibitors, and an anti-Trop2 antibody-drug conjugate (sacituzumab govitecan) for combined therapy will help more TNBC patients [125]. However, gastrointestinal side effects, myelosuppression, and impairment of liver function are among the negative consequences of PARP inhibitors in TNBC [126]. The majority of patients do not benefit from monotherapy with PD-L1 inhibitors [127]. Adverse effects of the anti-trop-2 antibody-drug combination include diarrhea (13%), anemia (14%), leukopenia (16%), and neutropenia (39%). Consequently, novel insights into CAR-T in solid tumors provide new avenues for TNBC treatment. Through preclinical or clinical trials, these TAAs have been validated. TanCAR-T cells that express many distinct CARs or CAR-NK cells that express a single CAR are examples of prospective targets. TAAs in TNBC can also activate these cells. TAAs that are highly expressed in TNBC are referred to as potential targets in other solid tumors. These TAAs can activate CAR-T cells that express a single particular CAR. While some research points to possible CAR targets for TNBC treatment, more work is required to verify their viability (Table 1).

**Table 1:** Applications of CAR-T therapy in TNBC

	Targets	Researches	Authors
1	CD22	On the cell membrane of TNBC cell lines (BT549 and MDA-MB-231), CD22 is expressed	[64]
2	CD44v6	CAR-NK cells with a CD44v6 target are efficient against TNBC and TME immunosuppression.	[66]
3	CD70	In MDA-MB-435 cells, TanCAR-T cells that target CD70 and B7-H3 significantly inhibit tumour growth.	[67]
4	EpCAM	In EpCAM-positive patients, the combined administration of TY-52,156 and EpCAM-targeted CAR-T cells has anticancer effects.	[77]
5	MUC1-C CAR-T cells targeting	MUC1-C on TNBC’s surface may encourage immunological evasion and cancer development.	[82]
6	FcgRI (CD64)	When combined with trastuzumab, CD64-targeted CAR-T cells exhibit strong antitumor efficacy against SKBR 3 cells that express HER2.	[39]
7	Nectin-2	Nutlin-3a combined with DNAM-1 CAR-NK cells could be a potential breast cancer treatment.	[85]
8	avb3-integrin	CAR-T cells that are specifically targeted by integrin avb3 recognise and eliminate MDA-MB-231, and they also secrete IFN-ϫ and IL-2.	[86]
9	avb6 integrin	On the avb6-positive TNBC cell line, integrin avb6-targeted CAR-T cells co-expressing CXCR 1 or CXCR 2 can have strong antitumor effects. MDA-MB-468	[87]
10	B7-H3	When paired with radiation therapy, B7-H3-targeted CAR-T cells enhance the therapeutic efficacy on the TNBC cell line, MDAMB-231.	[88]
11	B7-H4	On MDA-MB-468 cells, B7-H4-targeting CAR-T cells exhibit cytolytic toxicity.	[38]
12	CSPG4	Cytolytic toxicity of CSPG4-targeted CAR-T cells is observed on the TNBC cell line, MDA-MB-231.	[89]
13	FRα	MDA-MB-231 is a TNBC cell line that is treated as an antitumor by CAR-T cells that are directed against FR a.	[90, 91]
14	FAP	FAP-targeting CAR-T cells increase the anticancer activity in the TNBC cell line by eliminating cancer-associated fibroblasts (CAFs). HCC70	[92]
15	Fc_γ_RIII (CD16)	Combining cetuximab with CD16-targeting CAR-T cells causes TNBC cells to undergo apoptosis.	[93]
16	Fc_γ_RII (CD32A)	Combining cetuximab or panitumumab with CD32A-targeting CAR-T cells eradicates the MDA-MB-468 cell line.	[94]
17	ICAM1	TNBC cells expressing ICAM 1 are efficiently recognised by ICAM1-targeting CAR-T cells, which therefore stop their proliferation.	[95]
18	MSLN	MSLN-positive MCF 7 breast cancer cells can be specifically eliminated by CAR-T cells that target MSLN. For MDA-MB-231 xenografts, oncolytic adenoviruses that target TGF-b augment the antitumor effects of CAR-T cells that target MSLN.	[35] [96]
19	Nectin-4	CAR-T cells that target nectin-4 exhibit a time-dependent reduction in the within four hours, the MDA-MB-453 cell line’s cellular index	[97]
20	NKG2D	NKG2D-targeting CAR-T cells have antitumor activity against MDA-MB-231 and MDA-MB-468, two NKG2DL-positive TNBC cell lines.	[98]
21	PD-L1	The TNBC cell line, MDAMB-231, is cytotoxically affected by CAR-T cells that are directed against PDL1.	[36]
22	SSEA-4	Targeting SSEA-4, CAR-T cells had antitumor effects on the TNBC cell line MDA-MB-231, which expresses high levels of SSEA-4.	[100]
23	SLC3A2	SLC3A2-targeting CAR-T cells cause cytotoxicity in MDA-MB-231 and MDA-MB-468 cells.	[101]
24	TEM8/ANTXR1	CAR-T cells that specifically target TEM 8 have anticancer effects on MDA-MB-468, TNBC patient-derived xenografts (PDXs), and the TNBC cell line.	[102]
25	TROP2	Targeting Trop2 CAR-T cells causes the MDA-MB-231 cell line, which has strong Trop 2 expression, to exhibit targeted and potent cytotoxicity.	[103]
26	AXL	CAR-T cells directed against AXL demonstrate antitumor efficacy against AXL-positive MDA-MB-231 and MDAMB-468.	[37]
27	c-MET	The anticancer activity of c-Met-targeted CAR-T cells has been demonstrated against the c-Metpositive TNBC cell line, BT20, as well as the breast cancer cell line, TB129.	[104]
28	EGFR	Antitumor activity of EGFR-targeted CAR-T cells is demonstrated against MCF-7EGFR, MDA-MB-231, and SK-BR-3 cells. To increase the effectiveness against TNBC, EGFR-targeted CAR-T cells in combination with olaparib and Poly I: C block the recruitment of MDSCs. The combination of radiation therapy and EGFR-targeted T cells efficiently increases the death of TNBC cells.	[27] [105, 106] [107]
29	PTK7	PTK7-positive MDA-DB-468 is significantly cytotoxically affected by PTK7-targeted CAR-T cells.	[108]
30	VEGFR 2/3	VEGFR-2/3-targeted CART cells secrete IFN-g, TNF-a, and IL-2 in addition to cytotoxically attacking VEGFR-2 and VEGFR-3 positive breast cancer cells.	[109]
31	ROR1	TGF-b receptor signalling inhibition improves ROR1-targeted CAR-T cells’ anticancer activity against TNBC. Fi-CAR-T cells that target ROR1 increase the anticancer activity of ROR1-targeted CAR-T cells against TNBC by secreting anti-PD-1 scFv into the TME.	[110] [30, 111]
32	RON (MST1R)	MST1R may be a novel target antigen for breast cancer CAR-T cell therapy. 50% of human breast tumours have overexpression of MST1R, a prognostic biomarker.	[112] [113]
33	tmTNF-a	CAR-T cells targeting tmTNF-a exhibit effectiveness against MDA-MB-231 of tmTNFa, and this effectiveness is enhanced when combined with PD-1 mAb.	[114]
34	nfP2 × 7	Strong antitumor efficaciousness of CAR-T cells targeting nfP2 × 7 is demonstrated in xenograft animal models of prostate cancer and TNBC.	[115]
35	csGRP78	Human pancreatic cancer is successfully treated by csGRP78-targeted CAR-T cells in preclinical models. csGRP78 levels are upregulated in breast cancer cells that have developed tamoxifen resistance.	[116] [117]
36	CEA	M5A, hMN-14, and BW431/26 CAR-T cells have the ability to effectively lyse HEK293T cells that express CEA and release IFN-γ.	[118]
37	PSMA	Both BCSCs and TNBC cells express PSMA.	[121]
38	CLDN 6	For the treatment of solid tumours, the ongoing phase 1/2 BNT211-01 trial has validated the controlled safety and effectiveness of CAR-T cells targeting CLDN6.	[123]
39	TF	TNBC cells are directly killed by TF-targeted CAR-NK cells.	[127]
40	G2D	Excellent cytolytic activity can be demonstrated by GD2-targeted CAR-T cells against GD2-positive TNBC.	[34]

## The possible side effects of TNBC CAR-T treatment

Selecting appropriate target antigens is a crucial phase in CAR-T cell therapy for solid tumors. A CAR-T cell-mediated unfavorable event known as the “on-target off-tumor” effect happens when normal tissues contain a target antigen that is CAR-T misdirected toward., particularly essential organs, even at extremely low levels. Choosing target antigens with undetectable expression levels or those that are entirely missing from normal tissues is the best way to avoid this undesirable outcome. Due to the overexpression of each target antigen in malignant tissue cells as well as its presence in normal tissues, this strategy is incredibly impractical. As a result, academics have concentrated on strategies that may be useful for stopping or lessening the incidence of such incidents. To target TNBC, for example, CAR-Ts have been constructed with “safety switches” that incorporate suicide genes [128]. Furthermore, to prevent off-tumor toxicity, the use of synNotch and dual CARs in solid tumor CAR-T treatment has also been studied (134). ROR1-redirected CAR-Ts target both malignant cells that express ROR1 and stromal cells that express ROR1 (134). Bone marrow failure could be the consequence of this “on-target off-tumor” toxicity, hence an elaborate strategy is required to avoid this unfavorable outcome. ROR1-redirected CAR-Ts with synNotch receptors specific for EpCAM or B7-H3 were produced by Srivastava et al. (134). Lastly, these researchers observed that these CAR-Ts did not cause any damage while successfully preventing tumor growth [129]. Trans-signaling CARs may be unique in reducing on-target off-tumor toxicities in addition to these strategies (135). In the context of TNBC, Lanitis et al. produced trans-signaling FRa-redirected CAR-Ts. After that, these CAR-Ts were mixed to create two distinct CARs, one of which was directed against FRa and the other against mesothelin. The CAR co-stimulatory domain and CAR activation domain of these CAR-Ts were physically separated [130]. Preclinical evaluations of these CAR-Ts revealed that this approach may reduce the possibility of on-target off-tumor damage to normal tissues [130]. As mentioned earlier, studies have also been done on the value and efficacy of CARTs with affinity-tuned scFv targeting domains [131]. These CAR-Ts may aid in the differentiation of normal cells that generate the target antigen from tumor cells that overexpress it [131]. Thus, affinity-tuned ICAM-1-redirected CAR-Ts can target ICAM-1-overexpressing malignant cells while protecting ICAM-1-basal physiologically expressed normal cells, as demonstrated by Park et al. [131]. Furthermore, it has been proposed that mRNA-based CAR-Ts can be used as a strategy to prevent on-target off-tumor toxicities. Tchou et al. developed mRNA-based c-Met-redirected CAR-Ts to get CAR-mediated targeting of c-Met-expressing normal cells [132](137). They stated that both in vitro and in vivo, these cells demonstrated strong anticancer effectiveness against TNBC. The effectiveness and safety of these CAR-Ts were further assessed by these researchers in Phase I clinical studies (NCT01837602) [132]. One well-known adverse event of CAR-T cell therapy is the cytokine release storm (CRS), which is more common in patients with hematologic malignancies receiving CAR-T therapy [133]. The rapid activation of several immune mechanisms leads to CRS. Hyponatremia and cardiac-related toxicities are two possible significant harms. Patients receiving CAR-T treatment for solid tumors have also shown signs of CRS [133].

## CAR-T therapy clinical trials for TNBC

In order to overcome the safety and effectiveness issues brought on by the subtype heterogeneity of the illness, clinical trials for CAR-T-cell treatment in breast cancer must be carefully planned. There are several subtypes of breast cancer, including hormone receptor-positive, HER2-positive, and triple-negative breast cancer (TNBC). Each of these subtypes has distinct immunological profiles and molecular traits that can affect CAR-T-cell responses. To ensure a precise evaluation of CAR-T-cell efficacy in various tumor settings, trials must take this variation into consideration by classifying patients according to their subtype. Because breast cancer is close to healthy tissues, there is a greater chance of off-tumor damage, therefore safety concerns are especially crucial. While later stages assess efficacy objectives like progression-free survival and overall response rate, phase I trials frequently concentrate on dose-escalation to track toxicity levels. Furthermore, patients most likely to benefit from CAR-T-cell therapy may be identified with the use of trial designs that incorporate adaptive techniques, such as biomarker-driven cohorts and combination therapy arms with checkpoint inhibitors or other immunomodulators. Researchers can more accurately evaluate the promise of CAR-T-cell treatment for breast cancer by improving trial designs to address subtype variability and safety concerns [134].

Choosing the appropriate individuals for CAR-T-cell therapy in breast cancer is difficult but essential since it can greatly affect treatment results and reduce needless hazards. Finding predictive biomarkers or selection criteria is crucial because of the heterogeneity of breast cancer, which may cause certain patients to react to CAR-T therapy more than others. Tumor antigen expression levels (e.g., HER2, EGFR, MUC1) are potential biomarkers; these levels need to be high enough to guarantee CAR-T-cell binding and activation. Patients who are more likely to respond to CAR-T cells may also be identified by biomarkers that indicate an immune-supportive tumor microenvironment, such as increased PD-L1 expression or decreased levels of immunosuppressive cells (like Tregs or MDSCs), particularly when used in conjunction with immune checkpoint inhibitors. By signaling resistance or vulnerability to CAR-T-cell attack, genetic and epigenetic indicators such as specific gene mutations or immune-related gene expression profiles may also predict the effectiveness of CAR-T therapy. By improving CAR-T-cell persistence, lowering toxicity, and increasing overall efficacy, patient selection criteria based on these biomarkers could be optimized, leading to safer and more successful treatments [135].

One of the main concerns in clinical settings is managing the safety and side effects of CAR-T-cell therapy, especially cytokine release syndrome (CRS) and neurotoxicity. The symptoms of CRS, which range from a low fever to severe multi-organ failure, are caused by the CAR-T cells’ rapid and enormous release of cytokines upon activation. Neurological symptoms such as disorientation, seizures, or cerebral edema are signs of neurotoxicity, also known as immune effector cell-associated neurotoxicity syndrome (ICANS). In solid tumors like breast cancer, where CAR-T cells have a harder time entering the tumor microenvironment and may trigger longer or more intense immune responses, both CRS and ICANS provide significant dangers to patients. Monitoring techniques include routine evaluation of inflammatory markers that can indicate the early start of CRS, such as ferritin, C-reactive protein (CRP), and IL-6. The use of corticosteroids to treat severe inflammation or anti-cytokine therapy (such as tocilizumab for IL-6 inhibition) are interventions to reduce these risks. To improve safety profiles without sacrificing efficacy, preventive measures including employing “tunable” CAR designs with safety switches or dosing schedules that permit progressive CAR-T-cell activation are also being researched [136].

Because of the speed at which innovation frequently surpasses established frameworks, navigating the regulatory environment for CAR-T-cell therapies is difficult for both developers and regulatory bodies. Because CAR-T-cell treatments are highly customized and include genetic alterations unique to each patient, strict quality control is necessary to guarantee constant safety and effectiveness. Due to possible dangers like cytokine release syndrome (CRS), off-tumor toxicity, and delayed adverse effects, regulatory obstacles include strict requirements for proving long-term safety. Furthermore, the creation of CAR-T cells necessitates specific manufacturing procedures that adhere to Good Manufacturing Practice (GMP) guidelines, making scale-up initiatives more challenging. Regulatory agencies must update their rules to reflect recent developments in CAR-T, such as “off-the-shelf” allogeneic products and improved CAR designs, while striking a balance between patient safety and timely access. Establishing expedited approval pathways, such as adaptive trial designs and speedier approval processes, which could enable faster development without sacrificing strict control, requires cooperation between regulatory bodies, business, and academic institutions [137].

Concerns over equitable patient access are raised by the high prices of CAR-T-cell therapies, which frequently approach hundreds of thousands of dollars per treatment. The intricate, customized manufacturing process, which involves cell extraction, genetic alteration, expansion, and stringent quality testing—all of which call for specialized facilities and time-consuming procedures—is the source of the expenses. Because CAR-T-cell delivery and the management of associated toxicities, like cytokine release syndrome, need specialist medical teams and resources, these financial constraints are further exacerbated by the need for hospital infrastructure. These exorbitant costs render CAR-T-cell therapies unaffordable for many patients, especially those residing in low-income areas. Developing allogeneic “off-the-shelf” CAR-T products that do not require patient-specific cells, lowering manufacturing costs through automation and centralized production facilities, and implementing value-based pricing models are some of the options that must be investigated in order to overcome these obstacles. Broader access may also be supported by creative financing strategies like government subsidies and outcome-based reimbursement. To reach their full potential and offer fair treatment alternatives to all patient populations, it is imperative that these medicines be reasonably priced [138].

As was mentioned throughout the paper, there aren’t many clinical trials examining CAR-T therapy for the treatment of TNBC. Some of these trials are over, but others are still ongoing. Only a tiny portion of the finished trials have released their findings. Specifically, it was noted that the Phase I clinical trial (NCT02706392) assessing ROR1-redirected CAR-Ts in patients with different solid cancers, including metastatic TNBC, found that grade 1 CRS was present in half of the four TNBC patients [139].

A Phase I clinical trial (NCT01837602) evaluated the safety and efficacy of intratumoral delivery of c-Met-redirected CAR-Ts in patients with metastatic breast cancer. Findings showed that the CAR-Ts had a positive response and that there were no CAR-T-associated toxicities (> grade 1), as reported by Tchou et al. [132]. Furthermore, in a different Phase I clinical trial (NCT03060356) investigating the same target antigen [140], five patients experienced grade 1 or 2 CAR-T delivery-associated adverse effects (no grade 3 or CRS were noted).

For CRS, prompt clinical care is essential to prevent the illness from getting worse. When treating low-grade CRS, corticosteroids or antihistamines are usually recommended [133]. But improved ways are required when it comes to CAR-T therapy-mediated CRS, especially when it comes to the treatment of hematologic cancers like B-cell acute lymphoblastic leukemia (B-ALL) [141]. Several effective therapies for managing severe fatal CRS after CAR-T therapy include: hemofiltration, fractionated CAR-T infusion, antibody-based immunotherapy pretreatment, GM-CSF blocking, IL-1 and IL-6 inhibition, and therapeutic plasma exchange [142]. These strategies also apply to other solid tumor CAR-T therapies, such as TNBC CAR-T therapy. An overview of various clinical trials examining CAR-Ts against various target antigens for the treatment of solid tumours, including TNBC (Table 2).

**Table 2:** An overview of various clinical trials examining CAR-Ts against various target antigens for the treatment of solid tumours, including TNBC

Target antigen	ClinicalTrials.gov identifier	Phase	Participants	Source	Start-completion date
NKG2D ligand	NCT04107142	I	10	Allogeneic	2019–2021
ROR1	NCT02706392	I	21	Allogeneic	2016–2021
c-Met	NCT01837602 NCT03060356	I Early I	6	Allogeneic	2013–2018 2016–2020
Mesothelin	NCT01355965 NCT02580747 NCT02792114 NCT02414269	I I I I/II	77 18 20 186 113	Allogeneic	2011–2015 2015–2018 2016–2023 2015–2024
MUC1	NCT02587689	I/II	20	Allogeneic	2015–2018
A cleaved form of MUC1	NCT04020575	I	69	Allogeneic	2020–2035
TnMUC1	NCT04025216	I	112	Allogeneic	2019–2036

## TME’s impact on CAR-T therapy for TNBC

Because it prevents CAR-T-cell infiltration, persistence, and activity, the immune-suppressive tumor microenvironment (TME) in breast cancer poses a significant obstacle to the effectiveness of CAR-T-cell therapy. Immunosuppressive cytokines including TGF-β and IL-10 are secreted by TME components like tumor-associated macrophages (TAMs), regulatory T cells (Tregs), and myeloid-derived suppressor cells (MDSCs), which inhibit the function and proliferation of CAR-T cells. Furthermore, the TME’s hypoxic and nutrient-deficient settings can cause CAR-T-cell exhaustion, which further reduces the therapeutic potential of these cells. Immune checkpoint molecules like PD-L1 are frequently expressed by tumor cells themselves. These molecules interact with CAR-T cell inhibitory receptors to “turn off” the immune response. Researchers are investigating methods to reverse these effects, including designing CAR-T cells to withstand immunosuppressive signals, combining CAR-T cells with immune checkpoint inhibitors, and altering the TME using targeted medications that lower Tregs or MDSCs. These strategies seek to improve the conditions that allow CAR-T cells to proliferate and exhibit long-lasting anti-tumor effects [143].

**Fig. 4:**
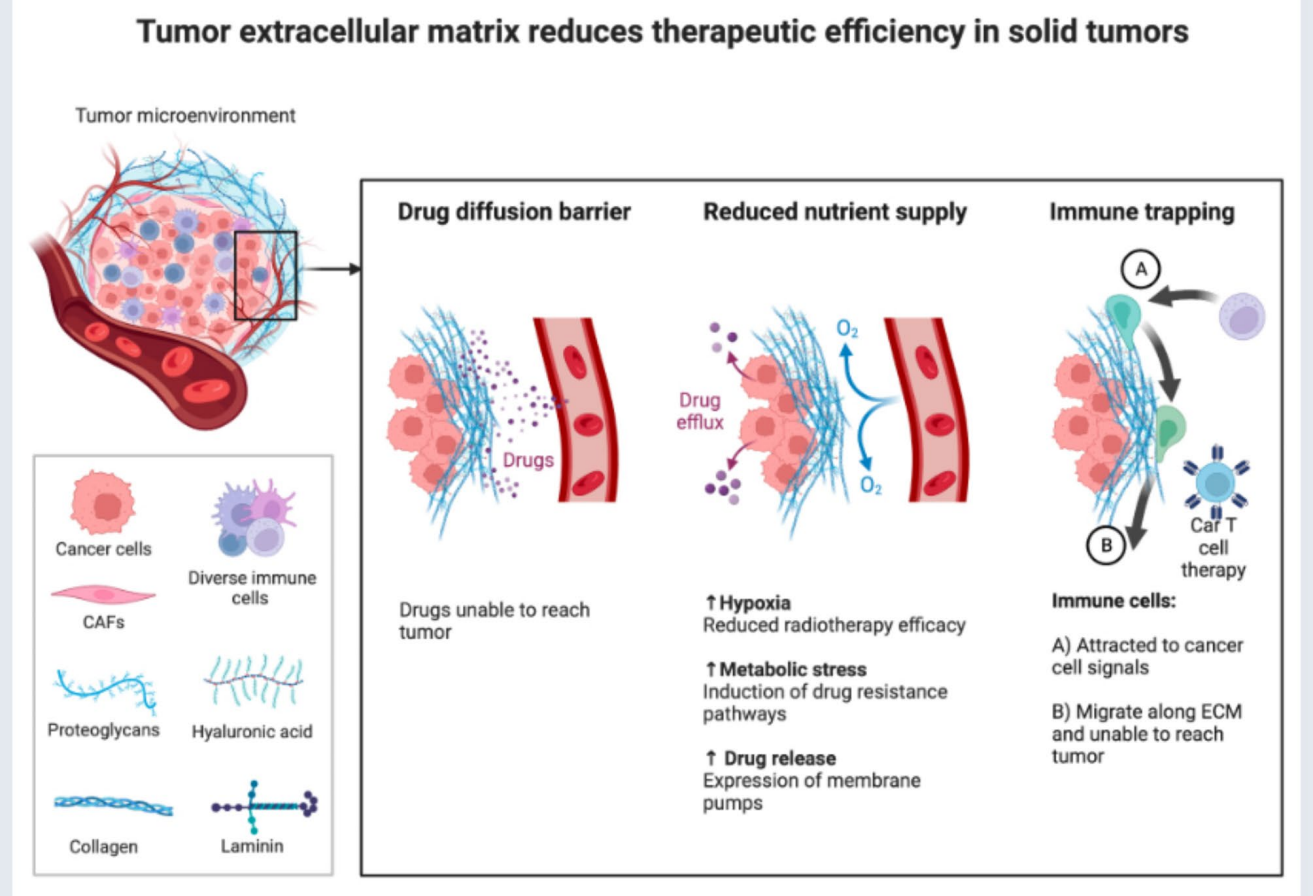
Tumor extracellular matrix reduces therapeutic efficiency in solid tumors. The tumor microenvironment (TME) comprises all components of a tumor. Of these components, the extracellular matrix (ECM) is the least well studied. Solid tumors induce high expression of ECM molecules (collagens, proteoglycans, hyaluronic acid and laminins), which become complex and disordered, resulting in altered characteristic. Here the ECM acts as a physical barrier, reducing the delivery of therapeutics, nutrients, and immune cells to solid tumors, and leading to poorer prognosis

The dynamic biological milieu made up of extracellular matrix (ECM), soluble materials, innate and adaptive immune cells, stromal cells, and signaling molecules is referred to as the “tumor microenvironment” (TME). TME is necessary for angiogenesis, tumor development, invasion, metastasis, immune evasion, and treatment-resistant tumors [144]. One feature of TME is hypoxia. The hypoxic area, composed of Treg cells, MDSCs, and TAMs, inhibits T-cell activation, proliferation, and cytotoxicity, which decreases the efficacy of the immune response to kill tumor cells [145] (Fig. 4). Research on treating cancers can benefit from focusing on TME components.

**Fig. 5:**
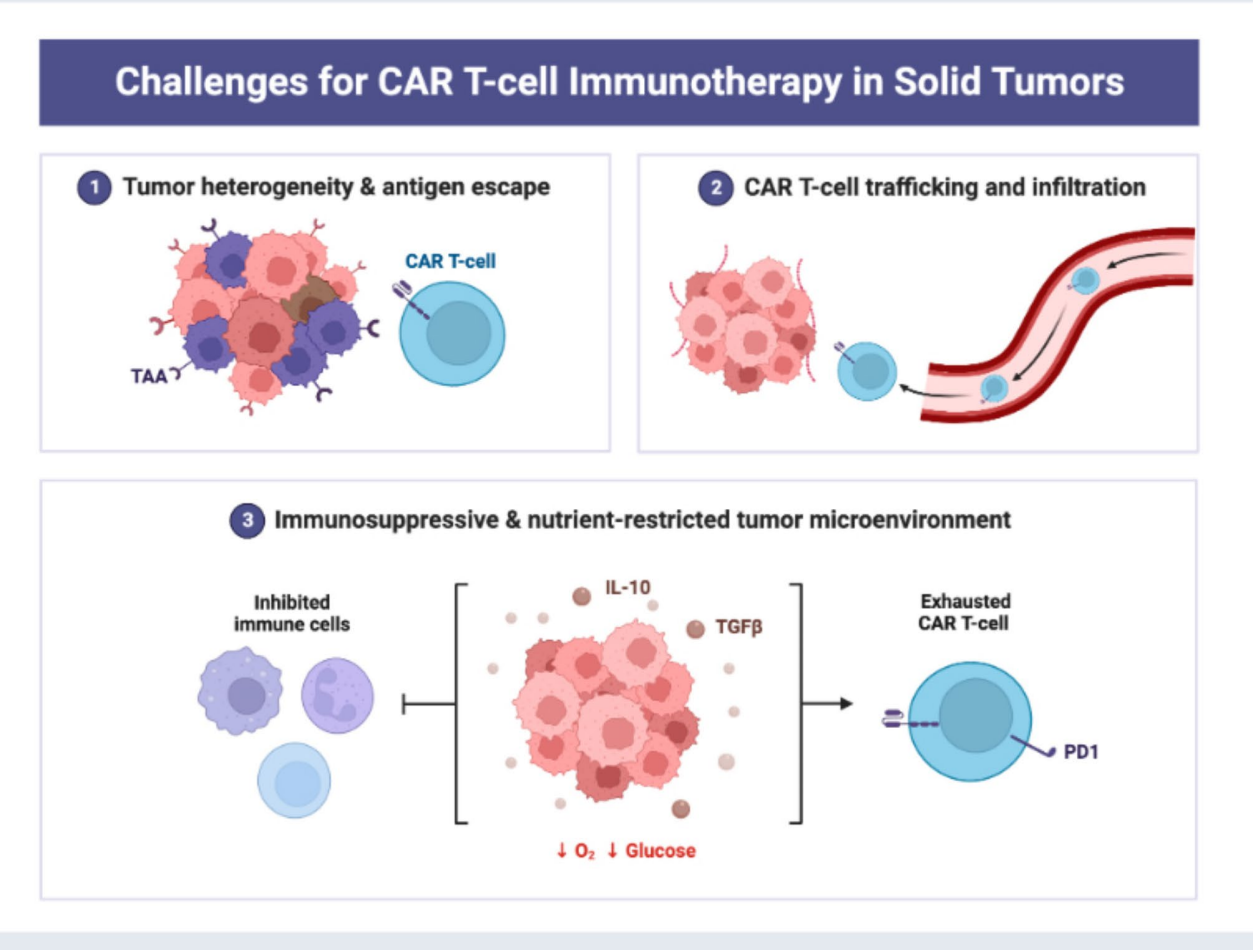
Challenges for CAR-T cell immunotherapy for solid tumour. Several challenges for CAR T cell immunotherapy for solid tumors are still existing including tumor heterogeneity, antigen escape, insufficient ability of CAR T cells for trafficking & infiltration of solid tumor environment, and the immunosuppressive & nutrients restrictive tumor environment

In the following sections, we address the role of tumor-associated macrophages (TAMs), regulatory T cells (Tregs), myeloid-derived suppressor cells (MDSCs), and cancer-associated fibroblasts (CAFs) in relation to CAR-T therapy in the treatment of breast cancer. Tumor growth, invasion, neoangiogenesis, inflammation, immunological suppression, and extracellular matrix remodeling are all significantly influenced by CAFs, or stromal cells, in the breast TME [146]. According to Wen et al., CAFs may facilitate tumor invasion in breast cancer cells that are integrin b3-positive. For CAFs, FAP is a therapeutic target in the TME of breast cancer that is HER2-positive [147]. Many studies have concentrated on CAFs in the TME to improve breast cancer therapy. Targeted immunotherapy against CAFs has been shown by Rivas et al. to overcome trastuzumab resistance in refractory HER2-positive breast cancers [148]. By eliminating CAFs, FAP-targeted CAR-T cells enhance the anticancer effect in TNBC in addition to treating HER2-positive breast cancers. Das et al. developed FAP-targeted CART cells to ablate CAF, stop MDSC recruitment, and promote T-cell infiltration. These actions improved the anticancer efficacy against TNBC [149]. Therefore, limiting CAFs in the TME can increase the range of possible uses for CAR-T cells in the treatment of breast cancer. FAP-targeted CAR-T cells treat HER2-positive breast cancers and improve TNBC’s anticancer effect by removing CAFs. FAP-targeted CART cells were created by Das et al. to ablate CAF, prevent MDSC recruitment, and encourage T-cell infiltration. These actions improved the anticancer efficacy against TNBC [149]. The limited success of CAR T-cell therapy in solid tumors can be accounted to many challenges, including: (1) the heterogeneous expression of tumor-associated antigens (TAA), leading to outgrowth of antigen-negative tumor variants; (2) inefficient trafficking of CAR T cells to tumor sites and (3) the metabolically hostile tumor microenvironment that includes the presence of immunosuppressive molecules (TGFβ, IL-10, etc.) and cells (T-regs, MDSCs, etc.) and can lead to CAR T-cell exhaustion (Fig. 5).

Thus, reducing CAFs in the TME may improve the application of CAR-T cells in the treatment of breast cancer. In order to prevent T cell activation and infiltration, which impacts the therapeutic efficiency of CAR-T cells, MDSCs are drawn to breast cancers. Poly I: Olaparib suppresses MDSCs via the SDF1a/CXCR4 axis and boosts the antitumor activity of CAR-T cell therapy, while CAR-T cells that target EGFR in conjunction with olaparib limit MDSC recruitment and increase their efficacy on TNBC [150]. TAMs are a particular type of cells that foster tumor growth in the TME of breast cancer. They can be pro- or anti-tumor M2-like (M2-TAM) or M1-like (M1-TAM) TAMs [139]. M2-TAMs are the main constituents of the stroma around breast tumors. Therapeutic targets for breast cancer may include TAMs. TAMs play a role in breast cancer cell proliferation, invasion, survival, angiogenesis, and metastasis [151]. For instance, Liu et al. demonstrated that in TAM-induced breast cancer cells, the natural substance emodin inhibited the development of cancer stem cells (CSC) and the EMT [152]. In the TME of TNBC, Meng et al. clarified the molecular function of PD-L1 in reversing TAM polarization towards the M2 phenotype, offering new treatment approaches for refractory TNBC [153].

Tregs are CD4 + T cells that secrete inhibitory cytokines and suppress T cell growth. They are primarily controlled by FoxP3 expression [154]. Tregs are intimately linked to the development, spread, and local invasion of breast cancer. Núñez et al. discovered a correlation between Treg accumulation and breast cancer patients’ invasion of breast cancer cells and their metastatic motility into draining lymph nodes [155]. Qiu et al. demonstrated a favorable correlation between CCL5 expression levels and the extent of axillary lymph node metastases in BC patients [156]. The production and maintenance of the immunosuppressive TME depend on Tregs. According to Bai et al., ANXA 1 enhanced Treg cell function and promoted the growth of breast cancer cells.

## Recent advances in breast cancer CAR-based immunotherapy for TNBC

Apart from CAR-T treatment, CAR-T can also be used to modify NK cells, macrophages, and mesenchymal stem cells (MSCs) for use as tumor-treating agents. Specifically, targeted CAR macrophages (CARMs) have been shown to produce greater antitumor effects against HER2-positive human chronic myeloid leukemia passage cells and CD19-positive ALL cancer cells in the treatment of hematological tumors [157]. Certain CAR-macrophages (CAR-Ms) have been shown to have antitumor effects in solid tumors, including GD2-expressing neuroblastoma, HER2-positive ovarian cancer cell line SKOV3, and GD2-expressing melanomas [158]. Therefore, CAR-NK, CAR-M, and CAR-MSCs are useful in the therapy of breast cancer.

CAR-M induces antigen-specific phagocytosis and tumor clearance based on the specificity of CAR [159], . Pro-inflammatory cytokines and chemokines expressed by CAR-Ms induce a pro-inflammatory TME, and convert M2 macrophages into a pro-inflammatory (M1) phenotype [160]. Treatment for breast cancer has improved thanks to CAR-Ms. According to Duan et al., 4T1 breast cancer-bearing mice showed anticancer effects from VEGFR-targeted CAR macrophages activated by TLR 4 or IFN-γ receptors [161]. Novel approaches to CAR-M-based breast cancer treatment may be provided by ongoing clinical trials on CAR-M targeting MSLN [162].

NK cells with genetic engineering can express CAR. Tumor cells can be precisely recognized and eliminated by these CAR-NK cells [163]. The CAR structure of CAR-NK cells consists of an intracellular activation domain, transmembrane domain, and extracellular antigen-binding region, just like that of CAR-T cells [164]. Different targets can be identified by CAR-NK cells to treat breast cancer because of the differences in the extracellular domain. Several forms of breast cancer have been effectively treated using CAR-NK cells that target CD44v6, HER2, TF, B7-H6, EGFR, and PD-L1 [165]. In TNBC, Raftery et al. showed that CD44v6-targeted CAR-NK cells had a strong anti-tumor effect [166]. Hu verified that TF can be used as a novel target for TNBC CAR-NK cell immunotherapy. L-ICON, an antibody-like immunoconjugate aimed at the TF, increased its effectiveness in vitro.

According to Lin et al., focusing on B7-H6 CAR-NK cells caused breast cancer cells resistant to fulvestrant to die [167]. CAR-NK cells are one of the treatment options for HER2-positive breast cancer. BT-474, SKBR 3, and MDAMB453 breast cancer cell lines that express HER2 were more resistant when CARNK cells were directed against HER2 [165]. Xia et al. found that, in contrast to HER2-targeted CAR-NK cells, HER2-targeted CAR-NK cells coexpressing sPD-1 demonstrated increased cytotoxicity against HER2-positive breast cancer cells with strong HER2 and PD-L1 expression [165]. HER2-targeted CAR-NK cells showed notable lethal efficacy even in the solid tumor microenvironment (TME) with concentrated immunosuppressive elements present. Human lung epithelial cells that physically express HER2 were not toxically affected by HER2-targeted CAR-NK cells, suggesting that these cells may be more helpful in the therapy of breast cancer [168].

The anticancer activity of CAR-NK cells may be impacted by the TME [169]. CAR-NK cells modify the TME by focusing on the matrix components. According to Fabian et al., TNBC was treated by NK cells that targeted PD-L1, which killed MDSCs [165]. CAR-NK cells have a good therapeutic effect on distantly located breast cancer metastases.

Chen et al. showed that CAR-NK cells that targeted EGFR prevented breast cancer brain metastases [170]. Thus, novel concepts and approaches for the clinical therapy of breast cancer are made possible by the advancements made in CAR-NK cell research.

MSCs improve healing, release cytokines, and are implicated in the immunological response [171]. Through the genetic engineering of expression-specific CAR, MSCs target distinct antigens, offering novel concepts and methods for treating cancer [172]. Despite the paucity of research on CAR-MSC therapy, indications point to a significant promise for treating breast cancer.

## Strategies to enhance CAR-T therapy efficacy for TNBC

In recent years, CAR-T therapy has emerged as a promising treatment option for hematologic malignancies, yet its efficacy in solid tumors, particularly in triple-negative breast cancer (TNBC), faces significant challenges [173]. TNBC, known for its resistance to standard therapies, presents an opportunity for immunotherapy-based interventions due to its immunogenic characteristics. The development and optimization of CAR-T therapy for TNBC require innovative strategies to overcome existing barriers and enhance treatment efficacy [173, 174].

Since CAR-T cells must continue to be active in the body for an extended period of time in order to continually target and eradicate tumor cells, maintaining their proliferation and persistence is crucial for long-term therapeutic efficacy in breast cancer. However, because of immunosuppressive signals and the severe circumstances in the tumor microenvironment, CAR-T cells in solid tumors frequently experience quick exhaustion and limited survival. Co-stimulation using extra signaling domains, like 4-1BB or CD28, which improve CAR-T cell activation, survival, and proliferation, is one way to increase CAR-T cell persistence. Maintaining CAR-T cell viability and encouraging in vivo expansion have also been demonstrated to be promising outcomes of genetic alterations that express cytokines such as IL-7 and IL-15. Furthermore, it has been investigated if adding “memory” T-cell traits to CAR-T cells can prolong their lifespan and maintain their functional potency over time. In solid tumor situations such as breast cancer, these strategies seek to maximize CAR-T-cell persistence, allowing for more robust and efficient responses [175].

To determine its place in future treatment regimens, it is essential to comprehend the long-term results of CAR-T-cell therapy in patients with breast cancer, including recurrence rates and overall survival. Although CAR-T-cell treatments have demonstrated remarkable outcomes in hematologic cancers, their use in solid tumors, such as breast cancer, is still in its infancy. Although there is a lack of long-term data, ongoing trials are being conducted to assess the overall survival, recurrence rates, and sustained efficacy of CAR-T cell treatment. Long-term reactions in breast cancer can be influenced by variables such tumor subtype, antigen expression, and the immunosuppressive tumor microenvironment. In some breast cancer subtypes, especially HER2-positive patients, early-phase studies indicate that CAR-T cells may provide long-lasting responses; nonetheless, the risk of relapse is still a worry because of the possibility of immune evasion or antigen loss. Long-term monitoring will be essential as CAR-T-cell treatments develop in order to evaluate long-term safety, including the possibility of late-onset toxicities, in addition to tumor control and survival. To assess the long-term benefits of CAR-T-cell therapy for breast cancer and to improve patient selection standards, ongoing observation and the creation of standardized follow-up procedures will be crucial [176].

### Combination therapies to improve CAR-T efficacy

Significant logistical obstacles arise from the intricacy and high expense of producing CAR-T cells, especially when expanding these treatments for broad clinical application. The manufacture of CAR-T cells is a rigorous, customized procedure that includes genetically altering and isolating a patient’s T cells, then going through a number of quality control and expansion stages. Although this individualized method is essential for effectiveness, it comes at a significant cost—often hundreds of thousands of dollars each treatment—and with lengthy production times. To guarantee cell viability from collection to injection, additional complications result from the requirement for specialized facilities, stringent regulatory compliance, and supply chain logistics. Simplifying the synthesis of CAR-T cells using automated, closed-system platforms and developing allogeneic “off-the-shelf” CAR-T products should lower production costs and duration, increasing the accessibility of therapies. Resolving these issues is essential for increasing access to a variety of patient populations and the wider therapeutic usage of CAR-T cells [177].

The effectiveness of CAR-T-cell therapy in treating breast cancer and other solid tumors may be greatly increased by combining it with other therapeutic approaches like immune checkpoint inhibitors, chemotherapy, or targeted medicines. In order to overcome the drawbacks of CAR-T-cell monotherapy, such as immune evasion or a non-permissive tumor microenvironment, combination therapies are justified. By preventing inhibitory signals, immune checkpoint inhibitors (such as anti-PD-1/PD-L1 or CTLA-4 inhibitors) can help rejuvenate worn-out CAR-T cells and increase their activity. By killing tumor cells and releasing neoantigens that CAR-T cells may target, chemotherapy may also have a synergistic effect. However, there are drawbacks to combining therapy, including as a higher chance of toxicity from over-activation of the immune system (such as worsening cytokine release syndrome or neurotoxicity) and possible consequences from drug interactions or tumor resistance mechanisms. It’s still crucial to strike a balance between the therapeutic window and making sure the two treatments work well together. Various combination techniques are being evaluated in ongoing clinical studies; preliminary findings indicate that these tactics can sometimes result in improved antitumor responses. However, additional optimization is required to improve safety profiles and determine the most advantageous combinations [178].

Combining CAR-T therapy with other treatment modalities holds promise in enhancing therapeutic outcomes for TNBC. Targeting components of the tumor microenvironment, such as cancer-associated fibroblasts (CAF) or extracellular matrix (ECM), can augment the tumoricidal effects of CAR-T cells. Additionally, the use of anti-angiogenic drugs or agents that target monocytes and macrophages can further potentiate the antitumor activity of CAR-T therapy in solid tumors. These combinations can help to mitigate the immunosuppressive environment and improve the efficacy of CAR-T cells. Thus, an integrated approach combining CAR-T therapy with other treatments offers a comprehensive strategy to tackle TNBC [173, 174].

### Novel effector cell types in CAR-T therapy

Exploration of alternative effector cell types expressing CARs, such as gd-CAR-T cells and CAR-expressing natural killer (NK) cells (CAR-NKs), offers potential solutions to overcome barriers in CAR-T therapy for TNBC. These engineered effector cells have shown promise in preclinical studies for both hematologic and solid cancers, including TNBC. By leveraging the unique properties of these effector cells, researchers aim to enhance the specificity, safety, and efficacy of CAR-T therapy in solid tumors. For instance, gd-CAR-T cells and CAR-NKs can target and eliminate cancer cells through distinct mechanisms, potentially reducing off-target effects and improving therapeutic outcomes. Additionally, these alternative CAR-expressing cells may overcome some of the immunosuppressive barriers within the tumor microenvironment, making them a valuable addition to the current CAR-T therapy landscape [139, 173, 179]. Continued research and clinical trials are essential to fully realize their potential and integrate them into standard cancer treatment protocols [180, 181].

CAR-T cells based on nanobodies are a novel immunotherapy strategy that takes advantage of the special qualities of nanobodies, which are tiny antibody fragments produced from camelid species (such as camels and alpacas). Compared to traditional antibodies, these nanobodies are more stable and smaller, which improves tissue penetration and gives them the ability to target antigens that larger antibodies might find harder to reach. The development of nanobody-based CAR-T cells aims to overcome a number of issues with conventional CAR-T treatments, including increasing T-cell activation efficiency, decreasing off-target effects, and boosting target specificity. In preclinical models, a number of studies have shown that employing nanobody-based CAR-T cells is feasible. These cells have exhibited encouraging anti-tumor effectiveness, especially in solid tumors that are often more challenging to treat with regular CAR-T cells. The safety, effectiveness, and long-term effects of these novel CAR-T designs are being evaluated in clinical studies; preliminary findings indicate that they may provide notable benefits, such as improved tumor targeting and decreased cytokine release syndrome (CRS). The potential of nanobody-based CAR-T cells to combat the immunosuppressive effects of the tumor microenvironment is also being investigated for use in solid tumors, such as breast cancer, as well as hematologic cancers [182].

### Advancements in target antigen selection

Improving the specificity of CAR-T therapy in solid tumors like TNBC requires careful selection of target antigens and addressing existing challenges. Novel approaches, such as engineering FcγRIII (CD16) and FcγRII (CD32)-chimeric receptors (CRs), enhance the therapeutic potential of CAR-T cells against TNBC. These CRs, combined with monoclonal antibodies targeting tumor-associated antigens, demonstrate the ability to eliminate TNBC cells through antibody-dependent cellular cytotoxicity (ADCC). Additionally, CD32-CR T cells can directly kill TNBC cells by recognizing alternative FcγR ligands on the cancer surface. These advancements increase the precision and effectiveness of CAR-T therapy, offering promising improvements in TNBC treatment. Continued research and clinical trials are essential to refine these technologies and optimize patient outcomes [183–185].

### Toxicity grading schemes for CAR T therapy of breast cancer

To comprehensively evaluate and control the possible side effects of this immunotherapy, toxicity grading systems for CAR-T cell therapy in breast cancer are crucial. Cytokine release syndrome (CRS) and immune effector cell-associated neurotoxicity syndrome (ICANS), the two main toxicities seen in CAR-T cell therapy, can both be mild to fatal. To standardize the evaluation of these toxicities, grading schemes like the ASTCT (American Society for Transplantation and Cellular Therapy) consensus grading and the Common Terminology Criteria for Adverse Events (CTCAE) have been widely used. Grade 1 denotes moderate symptoms that require minimal intervention, whereas Grade 4 denotes life-threatening symptoms that require extensive care. The ASTCT grading system, for instance, stratifies CRS based on clinical indications like fever, hypotension, and hypoxia. In a similar vein, ICANS is rated according to neurological evaluations, such as motor weakness, disorientation, and seizures, enabling doctors to respond with the proper interventions according to severity. When it comes to the therapeutic management of CAR-T cell treatments, these grading schemes are essential because they help determine whether to administer corticosteroids or tocilizumab to treat severe CRS or ICANS. These toxicity grading frameworks offer a crucial basis for assessing patient safety as CAR-T therapy spreads into solid tumors like breast cancer, allowing for more accurate monitoring and improving treatment results [186, 187].

## Conclusion

In recent years, CAR-T products have been made accessible as a treatment option for hematologic malignancies (R/R). However, there are also unexpected obstacles that severely limit the anticancer potential of these treatments when it comes to CAR-T-mediated targeting of solid tumors or particular hematologic malignancies. TNBC is a kind of mixed breast cancer that is mostly unresponsive to standard therapeutic methods. This tumor’s immunogenic characteristics have demonstrated the potential therapeutic outcomes of immunotherapy-based interventions.

For instance, because of its promising clinical outcomes when combined with napacol tablet, the US FDA approved atezolizumab, a checkpoint inhibitor, for the treatment of locally or metastatic advanced unresectable TNBC [188, 189]. Though CAR-T therapy for TNBC is still in its early stages, the field is still developing as its aim is to find appropriate and targetable TAAs, primarily in preclinical and early clinical stages. It is crucial to address a few significant tumor-related CAR-T treatment barriers in advance to overcome the difficulties of CAR-T therapy in triple-negative breast cancer. To make sure CAR-T therapy for solid tumors is safe and effective, several essential strategies need to be implemented. For solid tumors, combining CAR-T therapy with other therapies can enhance the therapeutic results [190].

Drug therapy that targets CAF or ECM, for instance, can be utilized to boost the effects of CAR-T on tumors. Moreover, CAR-Ts can be supplied more tumoricidal through the use of treatments that destroy monocytes or macrophages, or through the use of anti-angiogenic drugs [190]. Additionally, research has been done on the expression of CARs about various effector cell types. In light of this, gd-CAR-Ts and CAR-expressing NK cells (CAR-NKs) have been investigated as potential therapeutics for hematologic and solid cancers, including TNBC. These replacement CAR-expressing effector cells may be able to assist in overcoming various CAR-T treatment barriers [191, 192](181,182).

To effectively treat TNBC it is imperative to enhance the specificity, safety, and efficacy of CAR-T therapy in solid tumors by carefully choosing the best target antigens and resolving unmet restriction concerns. Many novel approaches have been put forth and used in both in vitro and in vivo research to improve the therapeutic potential of CAR T cells against solid tumors including TNBC. For example, FcγRIII (CD16) and FcγRII (CD32)-chimeric receptors (CRs) have been generated by replacing the single chain variable fragment (scFv) of the classic CAR with the extracellular CD16 or the extracellular CD32 both fused with the classic intracellular CAR signaling molecules such as CD28/CD3ζ chain. When given in combination with mAb directed against tumor-associated antigens (TAA) CD16-CR and CD32-CR T cells can eliminate breast cancer cells, including the TNBC cells in vitro by antibody-dependent cellular cytotoxicity (ADCC) [183–185]. Also, CD32-CR is a cytotoxic triggering molecule that can directly eliminate, in vitro and in vivo, TNBC cells by sensing alternative FcγR ligand(s) on the cancer cell surface [184].

## Data Availability

No datasets were generated or analysed during the current study.

## Abbreviations

ALL: Acute lymphoblastic leukemia
ANXA1: Annexin A1
ADCC: Antibody-dependent cellular cytotoxicity
L-ICON1: Antibody-like immunoconjugate
ABCB1: ATP-binding cassette sub-family B member 1
B-ALL: B-cell acute lymphoblastic leukemia
BC: Breast Cancer
CSC: Cancer stem cells
CAFs: Cancer-associated fibroblasts
CEA: Carcino-embryonic antigen
CCL5: C-C motif chemokine ligand 5
CD3ζ: CD3 zeta chain
CD44v6: CD44 containing variant exon v6
CSGP4: Chondroitin Sulfate Proteoglycan 4
CSPG4: Chondroitin sulfate proteoglycan 4
CARMs: Coactivator-associated arginine methyltransferase 1
CRS: Cytokine Release Syndrome
GD2: Disialoganglioside
DAP12: DNAX Activation Protein12
ENPP1: Ectonucleotide pyrophosphatase/phosphodiesterase 1
ESMA: Endogenous Signaling Molecule Activating
EGFR: Epidermal Growth Factor Receptor
EpCAM: Epithelial cell adhesion molecule
ER: Estrogen Receptor
ECM: Extracellular matrix
ERK: Extracellular signal-regulated kinase
FcγR: Fc gamma R
FAP: Fibroblast activation protein
FGFR4: Fibroblast growth factor receptor-4
FRα: Folate receptor alpha
GD3S: Ganglioside GD3 synthase
GM-CSF: Granulocyte-macrophage colony-stimulating factor
IL-2Rβ: IL-2 receptor β-chain fragment
ITAM: Immunoreceptor Tyrosine-based Activation Motif
ICOS: Inducible T-cell costimulator
ITGB6: Integrin beta-6
ICAM1: Intercellular adhesion molecule-1
IFN-γ: Interferon gamma
IL-1: Interleukin 1
IL-2: Interleukin 2
IL-6: Interleukin 6
JAK-STAT: Janus Kinases/Signal transducer and activator of transcription protein
KLRK1/NKG2D: Killer cell lectin like receptor K1
gp100: Melanocytic protein
MSLN: Mesothelin
MAPK: Mitogen-activated protein kinase
MEK: Mitogen-activated protein kinase kinase
MDSCs: Myeloid-derived suppressor cells
NK: Natural killer
NF-kB: Nuclear factor kappa B
PI3K/AKT: Phosphatidylinositol-3-kinase/Protein kinase B
PLCγ: Phospholipase C γ
PARP: Poly ADP-ribose polymerase
PR: Progesterone Receptor
PD-L1: Programmed death-ligand 1
PKC: Protein-kinase C
RAS: Rat sarcoma virus
ROR1: Receptor tyrosine kinase-like orphan receptor 1
RTK: Receptor tyrosine kinases
HER-2: Receptor tyrosine-protein kinase erbB-2
Tregs: Regulatory T cells
scFv: Single chain variable fragment
SDF1α/CXCR4: Stromal Cell-Derived Factor 1 Alpha/C-X-C Chemokine Receptor 4
synNotch: Synthetic Notch
TRUCK: T cells redirected for antigen-unrestricted cytokine-initiated killing
AXL: TAM family receptor tyrosine kinase
TanCAR-T: Tandem CAR-T
TLR 4: Toll-like receptor 4
TGFβ: Transforming growth factor-beta
MUC1: Transmembrane glycoprotein mucin 1
TNBC: Triple-negative breast cancer
TROP2: Trophoblast surface antigen 2
TEM8/ANTRX1: Tumor endothelial marker 8/Anthrax toxin receptor 1
TME: Tumor microenvironment
TAAs: Tumor-Associated Antigens
TAMs: Tumor-associated macrophages
VEGFR: Vascular endothelial growth factor
